# Colony co-founding in ants is an active process by queens

**DOI:** 10.1038/s41598-020-70497-x

**Published:** 2020-08-11

**Authors:** Serge Aron, Jean-Louis Deneubourg

**Affiliations:** 1 Evolutionary Biology and Ecology, Université Libre de Bruxelles, Brussels, Belgium; 2 Center for Nonlinear Phenomena and Complex Systems, Université Libre de Bruxelles, Brussels, Belgium

**Keywords:** Behavioural ecology, Animal behaviour

## Abstract

Cooperative breeding may be selected for in animals when, on average, it confers greater benefits than solitary breeding. In a number of eusocial insects (i.e., ants, bees, wasps, and termites), queens join together to co-create new nests, a phenomenon known as colony co-founding. It has been hypothesised that co-founding evolved because queens obtain several fitness benefits. However, in ants, previous work has suggested that co-founding is a random process that results from high queen density and low nest-site availability. We experimentally examined nest-founding behaviour in the black garden ant, *Lasius niger*. We gave newly mated queens the choice between two empty nesting chambers, and compared their distribution across the two chambers with that expected under random allocation. We found that queens formed associations of various sizes; in most instances, queens group together in a single chamber. Across all experiments, the frequency of larger groups of queens was significantly higher than expected given random assortment. These results indicate colony co-founding in ants may actually be an active process resulting from mutual attraction among queens. That said, under natural conditions, ecological constraints may limit encounters among newly mated queens.

## Introduction

Cooperative breeding is a social system in which organisms create communal nests, and it has evolved repeatedly in a range of taxa, including insects, fish, birds, and mammals^1–7^. In cooperative breeding, several adults engage in social behaviours that benefit both themselves individually and the group as a whole. This system may be selected for when ecological constraints (e.g., nest-site limitation, predation, parasitism, unpredictable resource availability) and competition greatly diminish the expected fitness payoff of solitary breeding. Cooperative breeding can result in greater nesting success because it enhances survival and reproduction, alloparental care, and/or collective nest defence^8–13^. Related individuals may nest together because they obtain fitness benefits, either directly or indirectly (i.e., via kin selection)^14^. Unrelated individuals may also nest together because they derive benefits arising from mutualism, reciprocity, and/or group selection^15–19^.

Ecological constraints on solitary breeding appear to be major drivers of collaborative colony founding in the four main groups of eusocial insects—ants, bees, wasps and termites^20–25^. In the majority of ant species, foundation of a colony is the deadliest phase of the life cycle because newly mated queens are exposed to predation, starvation, disease, competition, and adverse environmental conditions (e.g. desiccation). Colony founding events have a very high failure rate, as high as 99% in some species [^26–29^ and references therein]. Although new colonies are created by single queens (haplometrosis) in most ants, the process can involve multiple queens (pleometrosis) in several species^20,26,30^. Founding associations have been documented across a dozen genera from three different ant subfamilies^20^. Collaborative colony founding, hereafter referred to as colony co-founding, is usually carried out by unrelated queens; therefore, it is unlikely to have evolved as a result of indirect fitness benefits^20,26,28,30^.

In ants, colony co-founding enhances the productivity and success of incipient colonies because it increases queen survival during the early founding phase^31–33^; promotes faster brood development^31–39^; and boosts the size of the initial workforce, providing greater protection against intraspecific brood raiding, predation, and/or adverse abiotic conditions^32,36,37,40–43^. However, there is a cost associated with colony co-founding. In most species, the collaboration among queens is transient, and, after the first workers emerge, all but one of the queens are usually eliminated via queen fighting and/or culling by workers^20,42^. Co-founding a colony is therefore a risky endeavour: while the surviving queen will reap the full reproductive benefits of the colony, the defeated queens will have zero fitness. Thus, co-founding should be selected for when, on average, queens achieve higher fitness than they could have as solitary foundresses; conversely, it should be selected against when fitness benefits are significantly lower. Then, co-founding would result from random encounters when co-founding and solitary founding provide roughly equal benefits.

Although considerable attention has been paid to the benefits of colony co-founding in eusocial insects, the proximate factors underlying the phenomenon have remained largely unexplored. In particular, it is unclear whether co-founding results from a random process in which queens are simply tolerant of one another (i.e., there is neither attraction nor repulsion) or whether it results from attraction among queens. Studies have shown that group size increases with increasing queen density in some ant species^43–45^. However, whether or not such associations were random was unclear. A laboratory study of co-founding in the ant *Lasius pallitarsis* suggested that queen association resulted from random allocation, but mutual attraction and active co-founding could occur with large queen density^45^. In the tree-nesting ant *Crematogaster scutellaris*, the number of groups formed by queens under natural conditions did not differ from that expected based on random allocation^46^, suggesting that newly mated queens were not actively co-founding colonies. However, this study did not take into account spatial variation in nest-site availability or the density of newly mated queens.

**Figure 1:**
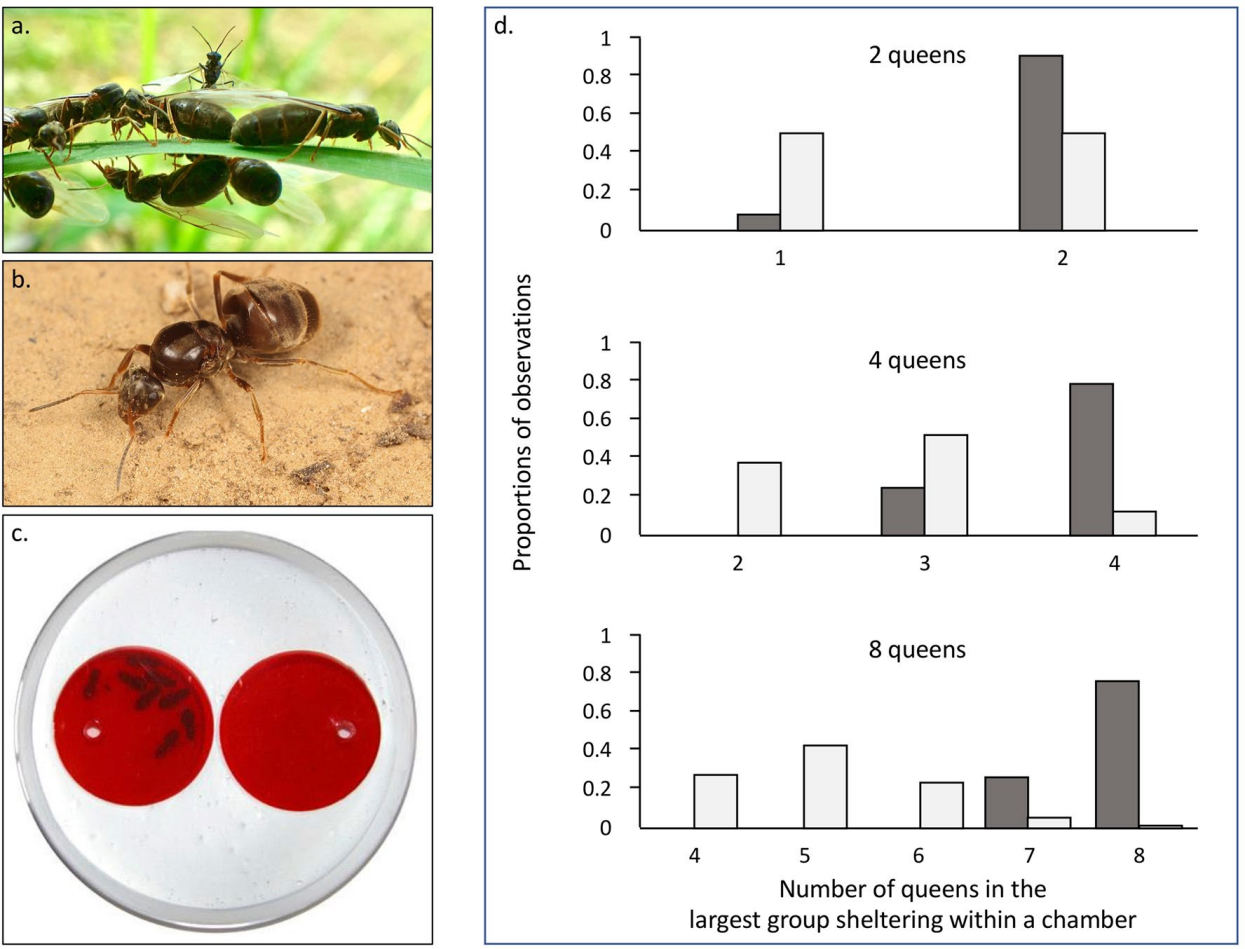
Grouping patterns of *L. niger* founding queens. (**a**) Virgin winged *L. niger* queens embarking on their mating flight from their nest of origin. In the centre of the image is a male standing on the wings of a queen. Picture: Q. Willot. (**b**) After mating, queens land and then lose or tear off their wings. They subsequently search for small burrows in the ground in which they found new colonies, either alone or with other queens. Picture: H. Darras. (**c**) Queen grouping during one of the experimental trials: 8 newly mated queens have clustered in a single nesting chamber. (**d**) Proportion of observations as a function of the number of queens in the largest group sheltering within a chamber (dark grey), and theoretical distribution (light grey) based on the assumption of random assortment (see “Methods”). Experimental trials were performed with *N* = 2 queens (*n* = 34), 4 queens (*n* = 23), and 8 queens (*n* = 25). For example, three situations were possible in trials with 4 queens: 2 queens in each chamber (2); 3 queens in one chamber and 1 queen in the other chamber (3); and all 4 queens in a single chamber (4). The graphs only show results for experimental trials in which *all* the queens were sheltered (i.e., none remained in the arena).

Here, we examined whether colony co-founding could result from queens actively grouping together. We used the black garden ant, *Lasius niger*, as a model system (Fig. 1a,b). In this species, mating occurs during large-scale nuptial flights, where thousands of sexuals from many colonies gather for a few hours. Once mated, queens land in an unknown environment, lose their wings, and quickly find a nesting site (small burrows in the open soil or under stones). In about 18–25% of cases, groups of 2–5 unrelated queens co-found colonies^42,47^. However, after the first workers emerge, queens start fighting with each other. Ultimately, only one queen survives, and she alone benefits from the colony’s future reproductive success. An experimental study of colony founding in *L. niger* offered newly mated queens an asymmetrical binary choice of nesting chambers: queens could settle either in an empty chamber or in a chamber containing another newly mated queen^42^. The study found that queens did not display a preference for either scenario, supporting the conclusion that colony co-founding was likely a random process promoted by high queen densities. To better understand the forces driving colony co-founding, we explored whether newly mated queens actively nested in groups. To this end, we presented newly mated queens with a symmetrical binary choice between two nesting chambers that were both initially unoccupied. We investigated how queen number affected the grouping patterns of queens across the two chambers by carrying out experimental trials involving two, four, and eight queens. Queens were allowed to move freely between the two chambers. We compared the observed grouping patterns of the queens across the two chambers after 24 h with the expected grouping patterns given random allocation based on stochastic simulations. Under conditions of random allocation, there would be no attraction among queens, and the queens would have an equal probability of ending up in either chamber (*p* = 0.5). If queens were actively grouping together, frequencies of larger groups of queens would be higher than expected based on random allocation. Conversely, if queens were actively avoiding each other, frequencies of larger groups of queens would be lower than expected based on random allocation.

## Results

In the three types of experimental trials (*N* = 2, 4, or 8 queens), the vast majority of queens ended up in one of the two chambers (Table 1 and Supplementary Table S1). The mean proportion of sheltered queens did not differ among trials (Kruskal–Wallis one-way analysis of variance, *P* > 0.05).

**Table 1:** Description of experiment and queen grouping patterns.

Experimental trial	*n*	# sheltered queens (S) (# trials)	Proportion of sheltered queens ± SD across all trials	% trials where queens sheltered in the same chamber	MWSE ± SD	*P*
2 queens	38		0.92 ± 0.24			
		2 (34)		91%	1.91 ± 0.28	< 0.0001
		1 (2)		–	–	–
		0 (2)		–	–	
4 queens	38		0.76 ± 0.36			
		4 (23)		78%	3.78 ± 0.41	< 0.0001
		3 (5)		60%	2.6 ± 0.49	< 0.034
		2 (2)		100%	2.0 ± 0.0	
		1 (4)		–	–	
		0 (4)		–	–	
8 queens	41		0.84 ± 0.26			
		8 (25)		76%	7.76 ± 0.43	< 0.0001
		7 (5)		80%	6.40 ± 1.20	< 0.0001
		6 (0)		–	–	
		5 (2)		50%	4.50 ± 0.50	
		4 (5)		60%	3.20 ± 0.98	
		3 (3)		66%	2.66 ± 0.47	
		2 (0)		–	–	
		1 (0)		–	–	
		0 (1)		–	–	

The table indicates the results of the different types of experimental trials (*N* = 2, 4, or 8 queens released in the arena at the start of the trial); the total number of trials of each type (*n*); the total number of sheltered queens (*S*) and the number of trials in which different values of *S* occurred (# trials); the mean proportion of sheltered queens ± SD across all the experimental trials of a certain type; the percentage of experimental trials in which sheltered queens were all in the same chamber; the mean number of sheltered queens in the largest group (MWSE) ± SD; and the probability of obtaining MWSE by chance, based on 10,000 simulations of random allocation outcomes*.* Experimental trials in which *S* = 0 or *S* = 1 were excluded when calculating MWSE and *P.*

First, we evaluated the results for the trials in which *all* the queens were sheltered (2 queens: 34/38 trials [89%)]; 4 queens: 23/38 trials [61%]; 8 queens: 25/41 trials [61%]; Table 1). Remarkably, queens grouped together in a single chamber in 91% of the trials with 2 queens (*n* = 34), in 78% of the trials with 4 queens (*n* = 23), and in 76% of the trials with 8 queens (*n* = 25) (Table 1). The mean proportion of sheltered queens found in the largest group was significantly greater than that expected given random assortment (*P* < 0.0001 for all three trial types; Fig. 1d).

Second, we evaluated the results for the trials in which some queens remained in the arena. After excluding trials with 0–1 sheltered queens, we were left with two situations: trials with 4 queens in which there were 2–3 sheltered queens (7/38 trials [18%]) and trials with 8 queens in which there were 2–7 sheltered queens (15/41 [37%]) (Table 1). As previously, the mean proportion of sheltered queens found in the largest group was significantly greater than that expected given random assortment (*P* < 0.034 and *P* < 0.0001 for trials with 4 and 8 queens, respectively).

## Discussion

We show that newly mated queens actively formed groups when given the choice between two empty nesting chambers. This suggests that colony co-founding in *L. niger* is an active process that results from mutual attraction among queens.

Our results contrast with those from the few previous studies that have examined colony co-founding in ants, which assumed that the phenomenon resulted from queens simply being drawn to the safety of an enclosed nesting place rather than being drawn by the presence of other queens [see “Introduction”^42,46,48,49^]. One study specifically stated that there was no attraction or repulsion between *L. niger* foundresses^42^. The discrepancy between their findings and our findings may stem from differences in methodology. In our study, queens were given a symmetrical choice between two empty nesting chambers. In contrast, in Sommer and Hölldobler’s study^42^, queens were given an asymmetrical choice: they could shelter in an empty chamber or in a chamber that already contained a queen. However, the study did not make clear how the latter queen was kept in the chamber or whether the potential retention method affected the queen’s behaviour. Moreover, the sample size was small, so the probability of making a type II error (wrongly failing to reject the null hypothesis of random allocation) was high. Another possibility is that queens from different populations differ in their colony founding strategies, as has been observed in the seed-harvester ant *Pogonomyrmex californicus*: in some populations, queens found colonies solitarily, whereas, in other populations, unrelated queens co-found colonies^30,50^. In the latter case, the associations persist as the colony matures, which means that colonies are headed by several reproductive queens (*i.e*., primary polygyny)^33^. Colony founding strategy is correlated with aggressiveness in *P. californicus* queens, and aggressiveness and tolerance phenotypes are strongly influenced by genetics^35,50–53^. Although it cannot entirely be excluded, this scenario seems unlikely in *L. niger* since (i) queens sampled in different parts of Europe have been observed to group together in the laboratory [e.g.^42,54–60^] and (ii) collaboration among queens is unstable and always transforms into intense fighting when the first workers emerge, a phase that only a single queen survives.

Our study was time limited and restricted to the grouping patterns of queens after 24 h. Clearly, additional studies should help decipher the mechanisms involved in the nesting choice of founding queens. Among these, is the probability for a queen to enter a chamber a function of the number of foundresses already present? Does the time spent searching for a shelter influence the probability for a queen to join other queens? What are the exact behavioural interactions among co-founding queens? Do queens move between shelters under laboratory or natural conditions and, if so, does the probability of leaving a shelter vary with the number of congeners in the same chamber? Also, the density of newly mated queens was probably much greater than in the field, a situation that increased the likelihood of queens clustering in the same chamber. However, our results clearly show that the queens’ grouping patterns were not random; they indicate that there was mutual attraction among queens.

In *L. niger*, colony co-founding has been shown to confer clear demographic advantages, since multiple-foundress colonies have a higher rate of worker production than do single-foundress colonies^47,55^. The creation of a larger workforce within a shorter time period presumably enhances colony survival under natural conditions. Altogether, these findings suggest that the low frequency of colony co-founding in *L. niger* in nature (18–25% of incipient colonies)^42,47^ is due to a lower likelihood of queens encountering each other. This encounter frequency could be diminished by low local densities of newly mated queens, high abundances of nest sites, and/or the need for queens to move into the first nest site they find to avoid desiccation or predation. In addition, the propensity of queens to co-found could depend on intrinsic factors, such as body weight or size, metabolic resources and, ultimately, the probability of surviving the conflict during reversion to single-queen colonies^20,45,54,61,62^. Joining behaviour indeed appears to be influenced by queen condition in the ant *Lasius pallitarsis*, where heavier queens are significantly more likely to join others than lighter queens, consistent with predictions of improved competitive ability^45^. So far, queen condition was however not shown to affect co-founding in the black garden ant^42^.

In short, and in contrast to previous studies, this study shows that colony co-founding in the black garden ant, *L. niger*, is an active process likely rooted in strong mutual attraction among newly mated queens. It is possible that the same is true in other Formicidae. Identifying the specific mechanisms mediating this mutual attraction may be challenging from a technical perspective, but they should be explored in future research.

## Methods

Newly mated and dealated *L. niger* queens were collected within a few hours of a large mating flight, after the queens had landed, in June 2011 in Brussels, Belgium. The next day, groups of *N* = 2 (*n* = 38 experiments), *N* = 4 (*n* = 38), and *N* = 8 (*n* = 41) queens were created via random assignment, then released into an arena (diameter: 9 cm) formed by a layer of plaster. Within the arena, at equal distances from the centre, were two identical circular nesting chambers (diameter: 3.5 cm, area: 9.62 cm^2^) (Fig. 1c). Both chambers were equally accessible. They were covered by transparent red filters (LEE Filters, absorption spectrum: 550–850 nm) that limited the light entering the chambers while still allowing the ants to be observed; a hole (diameter: 5 mm) was created in each filter so that queens could freely enter and exit the chambers. During the experiment, the temperature and relative humidity were 24 °C and 50%, respectively. The queens did not need to be fed during the experiment because colony founding is claustral in *L. niger* : queens seal themselves up in small burrows and rear their first brood by metabolising their wing muscles and stored fat.

After 24 h, we counted the number of queens in each chamber. In nature, queens that do not find a shelter within such a timeframe would most likely not survive.

### Queen grouping patterns

To define the expected frequencies of grouping patterns under conditions of random allocation, we performed stochastic simulations in which queens were distributed across the two chambers (see Supplementary Methods for a detailed description of statistical procedure). We were interested in the variable *S*, which was the number of sheltered queens (defined as queens that had taken shelter in a chamber). For example, in an experimental trial with 4 queens, *S* could be equal to 4 (all 4 queens were sheltered), 3 (3 queens sheltered, 1 queen in the arena), or 2 (2 queens sheltered, 2 queens in the arena) (Table 1). Experimental trials where *S* = 1 or *S* = 0 were discarded since they were not informative. These simulations made it possible to calculate the mean number of queens expected under random allocation in the larger group (this group can be in the left of right chamber) of sheltered queens, for the experimental trials with *N* = 2, 4 or 8 queens. Using this approach, we examined two sets of data. First, we considered the data from experimental trials in which all the queens were sheltered (*S* = *N*). For each sample (*N* = 2, 4 or 8 queens), we calculated the mean number of queens in the larger group. Then, 10,000 simulations with random allocation of queens across the two chambers were performed, and we calculated the proportion of means simulated that were equal to or higher than the experimental mean. The null hypothesis that the queens’ grouping patterns arose from random allocation was rejected when this proportion was ≤ 0.05. Second, we considered the data from the experimental trials in which some of the queens remained in the arena (*S* < *N*). In this case, the small sample sizes for each grouping pattern (see Table 1) made it impossible to compare observed and expected queen grouping patterns. We summed the number of queens in the larger group of sheltered queens across the experimental trials for the different values of *S*. Therefore, simulations were weighted based on the number of sheltered queens observed (*S*) (Supplementary Methods). Comparisons between the results of the simulations and those of the experimental trials were realized as described above.

### Ethics

Research on the species in this study does not require ethical licences. All experiments were performed in accordance with relevant guidelines and regulations in Belgium.

## Supplementary information

Supplementary Information 1.

## Data Availability

All the datasets supporting this article are included in the present work and the Supplementary Information.
