# Supplementary material for: Colony co-founding in ants is an active process by queens
Source: Sci Rep. 2020 Aug 11;10:13539. doi: 10.1038/s41598-020-70497-x (PMC7419493; doi:10.1038/s41598-020-70497-x)
Supplement: Supplementary file 1 — Supplementary Information 1. [file 41598_2020_70497_MOESM1_ESM.pdf]

Supplementary Information

Colony co-founding in ants — an active process by queens

Serge Aron[1*] and Jean-Louis Deneubourg[2]

[1] Evolutionary Biology and Ecology, Université libre de Bruxelles, Brussels, Belgium

[2] Center for Nonlinear Phenomena and Complex Systems, Université libre de Bruxelles, Brussels, Belgium

* email: saron@ulb.ac.be

1. Supplementary Methods: Stochastic simulations and statistical analyses

The random choice hypothesis assumes that queens settle in one of the two chambers independently from each other. Their probability (p) to shelter in the left or the right chamber is constant, and equal to 0.5. *S* corresponds to the total number of queens sheltered after 24h of experiments, and *j* is the number of individuals in the most occupied chamber. Thus, for *S*= 2, *j*= 1 or 2; for *S*= 4, *j*= 2, 3 or 4; and for *S*= 8, *j*= 4, 5, 6, 7 or 8.

The probability $\Psi(S,j)$ that the most occupied chamber contains *j* individuals is:

$$\Psi(S,j) = \frac{2S!}{j!\,(S-j)!}0.5^S, \qquad S \geq j > \frac{S}{2} \quad (1)$$

and further, if *S* is even:

$$\Psi\left(S,\frac{S}{2}\right) = \frac{S!}{(\left(\frac{S}{2}\right)!)^2}0.5^S, \qquad j = \frac{S}{2} \quad (2)$$

For example, using equations (1) and (2),
for *S*= 2

- the probability that the most occupied chamber contains 2 queens (*i.e.,* the two queens are in the same chamber) is $\Psi(2,2) = 0.5$
- and, the probability that the most occupied chamber contains 1 queen (*i.e*., one queen in each chamber) is $\Psi(2,1) = 0.5$

for *S*= 3

- the probability that the most occupied chamber contains the 3 queens (*i.e.,* the three queens are in the same chamber) is $\Psi(3,3) = 0.25$
- and, the probability that the most occupied chamber contains 2 queens (*i.e.,* two queens in one chamber and one queen in the other chamber) is

$$\Psi(3,2) = 0.75$$

Figure 1d (see text) gives the theoretical distributions Ψ *(S,j)* and the experimental distributions when all the tested queens (*N*) were sheltered (thus, *S*= *N*) for the three conditions *N*= 2, *N*= 4 and *N*= 8, as a function of the number of individuals in the most occupied chamber *j*.

We compared the observed distribution of queens in the two chambers with the theoretical distribution of queens expected under random allocation as follows.

First, we considered only experimental trials where all the queens were sheltered (as shown Figure 1, see text). A sample of T experiments were performed with *S* queens being

sheltered (e.g., with N= 4, all the queens were found sheltered in T= 23 experiments). T simulations were then performed with S queens. In our simulations, each queen randomly settles in one of the two chambers; the probability of a queen to join the left or right chamber is equal. A pseudo-random number generator was used to assign a chamber (left or right) to each queen. The mean number of queens in the most occupied chamber was then calculated over the T simulations. The same procedure was repeated 10,000 times, giving a distribution of 10,000 means of the number of queens in the most occupied chamber. We then calculated the proportion of simulated means being equal or higher than the experimental mean. The null hypothesis that the queens' grouping patterns arose from random allocation was rejected when this proportion was ≤ 0.05 (corresponding to the P-value).

Second, a similar procedure was used for the experimental trials in which some of the queens remained in the arena (see Table 1). However, due to the small number of trials with such outcome, all trials with the same number of tested queens (N) were pooled. For example, in experiments with 4 tested queens, 5 trials resulted in 3 queens being sheltered (and 1 queen remaining in the arena), and 2 trials resulted in 2 queens being sheltered (and 2 queens remaining in the arena). Experimental trials where S= 1 or S= 0 were discarded since they were not informative. The mean number of queens in the most occupied chamber over the 7 simulations was calculated (5 simulations with S=3 sheltered queens and 2 simulations with S=2 sheltered queens). Then, we performed 10,000 repetitions of the 7 simulations. The P-value was calculated as in the previous situation.

2. Supplementary Table S1: Raw data

N	Nb. queens outside	Nb. queens in left chamber	Nb. queens in right chamber	*N*	Nb. queens outside	Nb. queens in left chamber	Nb. queens in right chamber	*N*	Nb. queens outside	Nb. queens in left chamber	Nb. queens in right chamber
**2**	0	2	0	**4**	0	4	0	**8**	0	8	0
**2**	0	2	0	**4**	0	4	0	**8**	0	8	0
**2**	0	2	0	**4**	0	4	0	**8**	0	8	0
**2**	0	2	0	**4**	0	4	0	**8**	0	8	0
**2**	0	2	0	**4**	0	4	0	**8**	0	8	0
**2**	0	2	0	**4**	0	3	1	**8**	0	8	0
**2**	0	2	0	**4**	0	3	1	**8**	0	8	0
**2**	0	2	0	**4**	0	3	1	**8**	0	8	0
**2**	0	2	0	**4**	0	1	3	**8**	0	8	0
**2**	0	2	0	**4**	0	1	3	**8**	0	8	0
**2**	0	2	0	**4**	0	0	4	**8**	0	8	0
**2**	0	2	0	**4**	0	0	4	**8**	0	8	0
**2**	0	2	0	**4**	0	0	4	**8**	0	7	1
**2**	0	2	0	**4**	0	0	4	**8**	0	7	1
**2**	0	2	0	**4**	0	0	4	**8**	0	1	7
**2**	0	2	0	**4**	0	0	4	**8**	0	1	7
**2**	0	2	0	**4**	0	0	4	**8**	0	1	7
**2**	0	2	0	**4**	0	0	4	**8**	0	1	7
**2**	0	1	1	**4**	0	0	4	**8**	0	0	8
**2**	0	1	1	**4**	0	0	4	**8**	0	0	8
**2**	0	1	1	**4**	0	0	4	**8**	0	0	8
**2**	0	0	2	**4**	0	0	4	**8**	0	0	8
**2**	0	0	2	**4**	0	0	4	**8**	0	0	8
**2**	0	0	2	**4**	1	3	0	**8**	0	0	8
**2**	0	0	2	**4**	1	2	1	**8**	0	0	8
**2**	0	0	2	**4**	1	1	2	**8**	1	7	0
**2**	0	0	2	**4**	1	0	3	**8**	1	7	0
**2**	0	0	2	**4**	1	0	3	**8**	1	7	0

**2**	0	0	2	**4**	2	2	0	**8**	1	4	3
**2**	0	0	2	**4**	2	0	2	**8**	1	0	7
**2**	0	0	2	**4**	3	1	0	**8**	3	5	0
**2**	0	0	2	**4**	3	1	0	**8**	3	4	1
**2**	0	0	2	**4**	3	1	0	**8**	4	4	0
**2**	0	0	2	**4**	3	0	1	**8**	4	4	0
**2**	1	1	0	**4**	4	0	0	**8**	4	2	2
**2**	1	1	0	**4**	4	0	0	**8**	4	2	2
**2**	2	0	0	**4**	4	0	0	**8**	4	0	4
**2**	2	0	0	**4**	4	0	0	**8**	5	3	0
								**8**	5	3	0
								**8**	5	1	2
								**8**	8	0	0

N: Number of queens released into the arena.
